# Effects of Different Positions and Angles of Implants in Maxillary Edentulous Jaw on Surrounding Bone Stress under Dynamic Loading: A Three-Dimensional Finite Element Analysis

**DOI:** 10.1155/2019/8074096

**Published:** 2019-12-17

**Authors:** Xiaqing Liu, Fang Pang, Ying Li, Hui Jia, Xiaohua Cui, Yuan Yue, Xuelian Yang, Qi Yang

**Affiliations:** ^1^Oral Multi-Disciplinary Treatment Center, The First Hospital of Shanxi Medical University, Taiyuan 030001, China; ^2^Shanxi Medical University School and Hospital of Stomatology, Taiyuan 030001, China

## Abstract

**Purpose:**

To evaluate the effects of different placements of mesial implants and different angles of distant implants in maxillary edentulous jaws on the stress on the implant and the surrounding bone tissue under dynamic loading.

**Materials and Methods:**

Cone beam computed tomography was used to acquire images of maxillary edentulous jaws. Using Mimics 17.0, Geomagic, and Unigraphics NX8.5 software, three-dimensional models were established: two mesial implants were placed vertically in the anterior region of the maxilla (bilateral central incisor, lateral incisor, and canine), and two distant implants were placed obliquely in the bilateral second premolar area at different inclined angles (15°, 30°, and 45°). The established models were designated I–IX. The models were subjected to dynamic load using Abaqus 6.12, with the working side posterior teeth loading of 150 N and simulation cycle of 0.875 s.

**Results:**

During the second to fourth phases of the mastication cycle, the stress was mainly concentrated on the neck of the distal implant. The stress of the distal implants was greater than that of mesial implants. Stress levels peaked in the third stage of the cycle. The stress of the distal cortical bone of distal implant of Model I reached the maximum of 183.437 MPa. The stress of the distal cortical bone and cancellous bone of distal implant of Model VIII represented the minima (62.989 MPa and 17.186 MPa, respectively).

**Conclusions:**

Our models showed optimal stress reductions when the mesial implants were located in the canine region and the distal implants tilted 30°.

## 1. Introduction

Economic and social development has accelerated the overall aging of the population, so dental prostheses have become necessary for improving the quality of life. Implant-based edentulous jaw restoration is becoming more and more important in dentistry. Compared to traditional complete dentures, implant restoration can more effectively improve oral/jaw function in patients with edentulous jaws, thus visibly improving their quality of life. Furthermore, the clinical effect is satisfactory and the long-term success rate is higher [1–5]. For edentulous patients, Malo [6] first put forward the concept of “All-on-4” in 2003, which states that even if four implants were used to complete the immediate restoration of an edentulous single jaw under reasonable distribution conditions, the implantation direction should include two vertical implants in the anterior jaw bone and/or two oblique implants (no more than 45°) in the posterior jaw bone. Oblique implants have the following advantages: the ability to reduce or even avoid incremental bone surgery [7]; greater initial stability for longer implants; and a shorter cantilever beam that prevents stress concentration. Given these advantages, work by Soto-Penaloza et al. documented a survival rate of implants surviving over 24 months of 99.8% [8].

However, because the biomechanical behavior of implanted dentures is different from the natural tooth support bridge and they lack periodontal ligaments and other soft tissue structures, implants cannot sense the occlusal force of teeth; this condition can easily lead to excessive load and subsequent restoration failure. Notably, implant design is a factor affecting bone stress distribution [9], indicating that the reasonable design of implants, during which biomechanical design should be factored, will be key to the implant's success [10, 11]. The three-dimensional (3D) finite element method is effective for studying implant biomechanics [12–14]. Currently, the analysis of All-on-4 with the 3D finite element method has become of great interest. Indeed, the number, location, and characteristics of implants, as well as the angle of implantation and the cantilever beam, have all been studied. Ozan and Kurtulmus-Yilmaz [15] evaluated the effect of implant inclination (0, 17, 30, and 45° angles) on stress distribution in the mandibular cortical bone and implant via 3D finite element static analysis. They showed that stress distribution improved when tilting the implants 30° or 45° posteriorly and using shorter cantilever lengths. Saber et al.[16] compared the amount and distribution of stress in the maxillary bone surrounding the implants between the All-on-4 method and a frequently used six-implant technique using different numbers and inclination angles. Four distal implants were placed and inclined 0, 15, 30, and 45 degrees, respectively. They found that increasing the inclination in posterior implants resulted in reduced cantilever length and maximized stress reduction in both the cancellous and cortical bones.

One issue with the above studies is their use of a static loading force at the contact point, which is significantly different from the dynamic loading force generated by actual masticatory movement. Chewing movement of the oral cavity is a dynamic process but is also cyclic and repeatable. To simulate the actual masticatory process more realistically, the bite force applied to the denture must change rapidly and dynamically with time [17, 18]. However, dynamic loads may further increase stress, thus increasing the likelihood of implant failure in the clinical environment; despite this risk, very few studies have analyzed edentulous jaw implants under dynamic loading. Here, we used dynamic loading [19–21] to model the effects of implant position and angle on bone stress around maxillary edentulous jaw implants at different stages of mastication. The results will provide clinicians with the optimal placement and angle of implantation for successful edentulous implant restoration.

## 2. Materials and Methods

This study and its methods were approved by the Ethics Committee of the First Affiliated Hospital of Shanxi Medical University (Ratification No. (2015) No. (Y10)).

### 2.1. Conical Beam CT Data

In the Department of Stomatology of the First Affiliated Hospital of Shanxi Medical University, a cone beam CT scanner (NewTomVGi, QRSRL, Italy) was used to screen imaging data for the following inclusion criteria: maxillary edentulous jaw, male, age 45–65 years, with maxillary shape integrity, maxillary height ≥15 mm, width ≥7 mm, and bone mineral density of 350–850 Hu. The model bone belongs to the D3-type bone classified by Misch [22]. Once a patient's imaging data met the inclusion criteria, that case was selected, the patient was contacted, informed consent was signed, the maxillary complete denture was copied, the developer was sprayed, and the cone beam CT was used once more to scan the maxillary resin denture.

### 2.2. Three-Dimensional Digital Modeling of the Maxilla

3D modeling of the maxilla was accomplished by transferring 2D images acquired by the CT scanner to 3D engineering software via Mimics 17.0 (Materialise's interactive medical image control system; Materialise, Belgium), an image processing tool that can compile 2D DICOM-formatted (CT/MRI) images and import them to 3D engineering applications.

In accordance with the image segmentation method described previously [23], CT images were imported into Mimics; then regional segmentation was performed and tissue thresholds were set to isolate and extract bone tissue. The model was a refined optimization model created in reverse-engineering software (Geomagic Studio 12.0, Geomagic, United States) as a 3D digital maxillary edentulous jaw model.

### 2.3. 3D Solid Models of Implants, Upper Supports, and Resin Teeth

**Figure 1:**
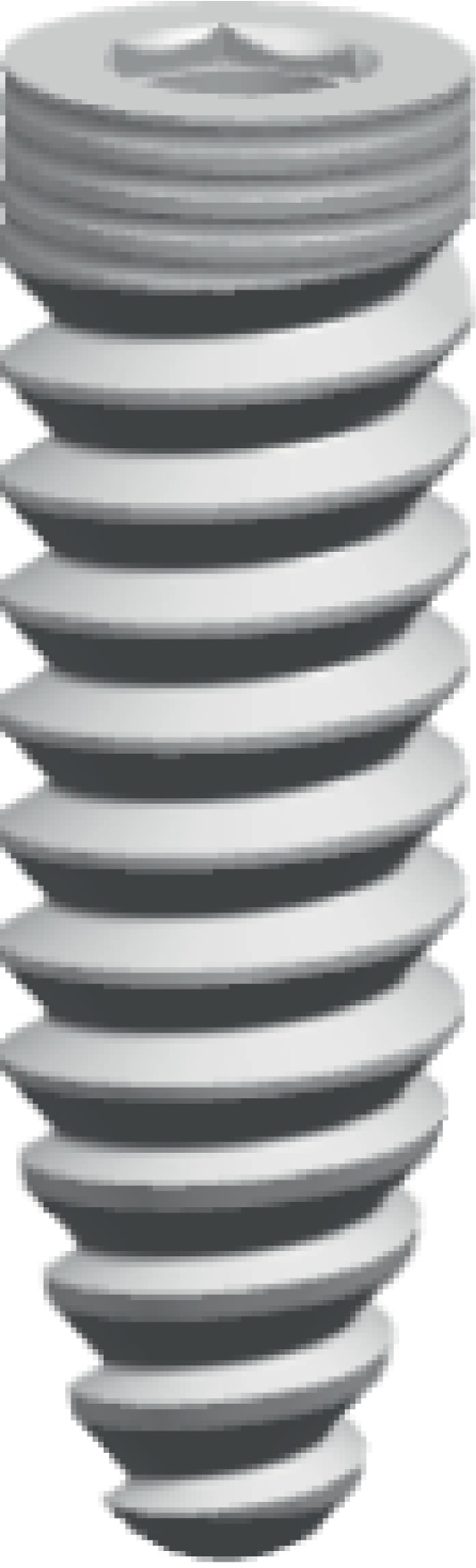
Three-dimensional model of the XIVE implant.

**Figure 2:**
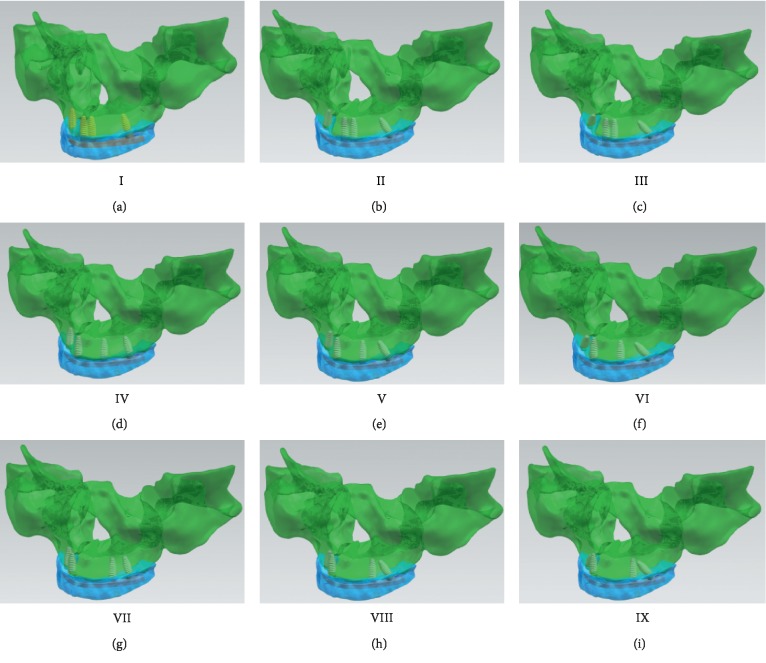
Three-dimensional solid models of the maxilla. Models used two mesial and two distal implants, a titanium upper bracket, and resin teeth. Model numbers represent different positions of mesial implants (I–III, IV–VI, and VII–IX for central incisor, lateral incisor, and canine, respectively) and different angles of distal implants (I/IV/VII, II/V/VIII and III/VI/IX for 15°, 30°, and 45°, respectively).

A solid 3D model of the implant was developed using the implant parameters (XIVE, Dentsply, Germany) in UG NX8.5 (Siemens PLM software, USA). Implants with different sizes and diameters can be modeled depending on jaw characteristics [24]. The following implant parameters were selected: diameter of 4.50 mm, length of 13.00 mm, pitch of 0.84 mm × 2.00 mm, thread thickness of 0.84 mm, thread height of 0.50 mm, abutment height of 4.00 mm, abutment taper of 6° (Figure 1). Two mesial implants were placed vertically in the anterior region of the maxilla (bilateral central incisor, lateral incisor, and canine), and two distal implants were placed obliquely in the bilateral second premolar area at different inclined angles (15°, 30°, and 45°) as described previously [15]. Nine models were defined (I–IX) (Figure 2). Model groups I–III, IV–VI, and VII–IX had central incisor, lateral incisor, and canine mesial implants, respectively; within each model group, the first, second, and third model had 15°, 30°, and 45° distal implants, respectively (e.g., model IX had canine mesial and 45° distal implants).

The upper bracket was modeled with pure titanium with a height of 5 mm, a width of 5 mm, and a bilateral end cantilever of 10 mm, which were consistent with the arc of the jaw bone and a fixed connection with the implant. To ensure equal lengths of the cantilever beams, the two distal implants had the same abutment position. Tooth arrangement was performed on the upper bracket using resin teeth and data acquired by cone beam CT; the teeth were arranged to the bilateral first molars as described previously [15].

### 2.4. Three-Dimensional Finite Element Modeling of the Maxilla

The 3D finite element model of the maxilla was established using the 3D finite element analysis method with Abaqus 6.12 (HKS, USA). Using previous research by Munari et al. [25, 26], the 3D model was grid optimized and exported from the Mimics software in unit format (element). The cell grid file was imported into Abaqus, and the unit type was set as the C3D10 three-dimensional tetrahedron element. Finally, we converted the mesh of the face into the mesh of the body. The numbers of cells in Models I–IX were 176984, 179846, 170268, 173691, 179023, 170566, 175505, 178491, and 170654, respectively; the numbers of nodes were 65389, 63710, 60354, 63059, 66190, 63242, 64210, 65748, and 68978, respectively.

**Figure 3:**
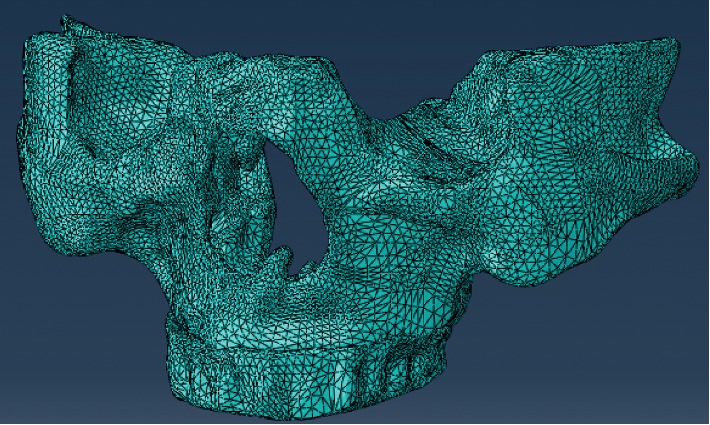
A representative three-dimensional finite element digital model of the maxilla with four implants, upper bracket, and resin teeth.

The gridded model was reintroduced to the Mimics software to set the reference values [27, 28] for the modulus of elasticity (MPa) of implants and pure titanium scaffolds (110000), denture (resin; 3520), cortical bone (13700), and cancellous bone (1370), as well as their Poisson ratios (0.35, 0.4, 0.3, and 0.3, respectively). The final model is shown in Figure 3.

### 2.5. Determination of Dynamic Load and Stress

The interface between the implant and bone was set to be 100% fully bonded in order to simulate the ideal osseointegration [19, 29]. The masticatory muscle effect was simulated by the constraint method in place of the masticatory muscle attachment. The surface was fixed, the degree of freedom at the upper end of the constraint was set to 0, and the full degree of freedom was restricted in three directions (*X*, *Y*, *Z*). Masticatory force is generally 100–200 N. In this study, 150 N was set as the working lateral posterior tooth load [19, 29]. A dynamic load period was set to 0.875 s, and the left side of the lateral movement was set as the working side. A dynamic load cycle of the posterior teeth (the simulated masticatory cycle) is divided into five stages, detailed in Table 1. Briefly, from 0.00 s to 0.13 s (stage 1), there is no loading force; from 0.13 s to 0.3 s (stages 2–4), 150 N loading force is modeled for each stage, although the loading positions (occlusal contact points) are different for each stage; from 0.3 s to 0.875 s (stage 5), the jaw is opening and there is no loading force [30].

## 3. Results

### 3.1. Stress Cloud Diagrams of Implants and Maxillary Models

**Figure 4:**
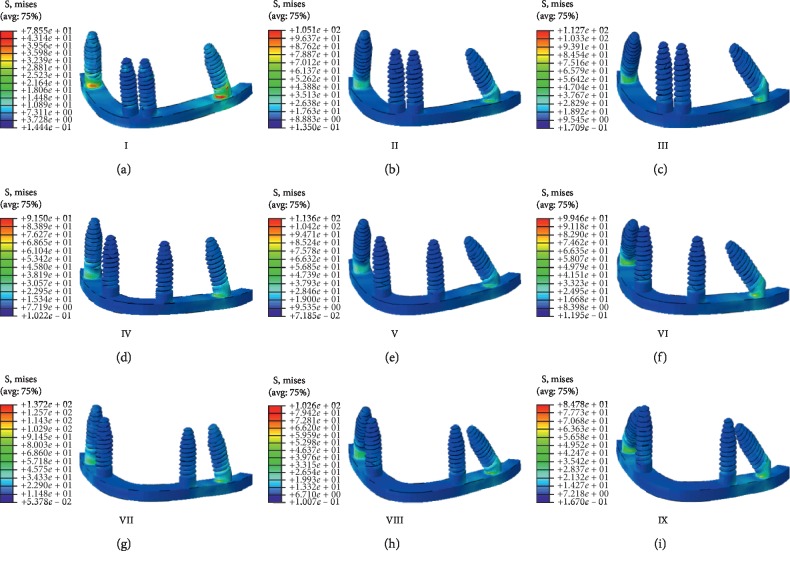
Stress distribution and intensity heat map of mesial and distal implants in the nine finite element models in dynamic loading stage 3. (a) Model I. (b) Model II. (c) Model III. (d) Model IV. (e) Model V. (f) Model VI. (g) Model VII. (h) Model VIII. (i) Model IX.

**Figure 5:**
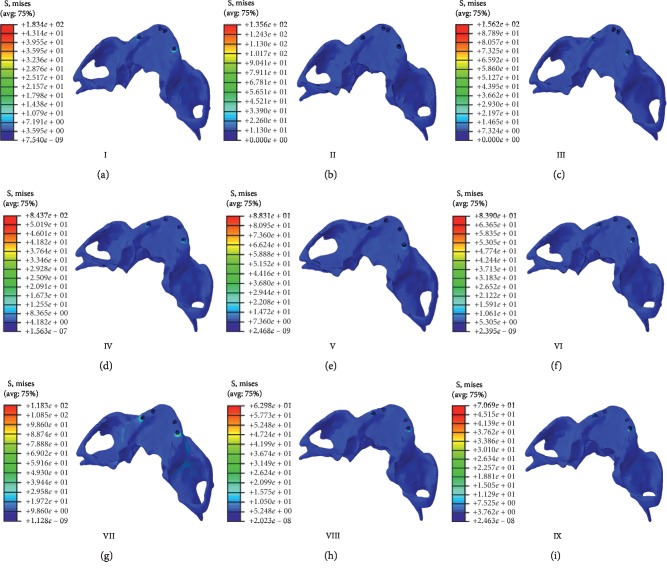
Stress distribution and intensity heat map of the maxilla in the nine finite element models in dynamic loading stage 3. (a) Model I. (b) Model II. (c) Model III. (d) Model IV. (e) Model V. (f) Model VI. (g) Model VII. (h) Model VIII. (i) Model IX.

All nine implant models produced equivalent stress cloud diagrams (Figure 4) in which the stress was mainly concentrated on the neck of the distal implant and gradually decreased to the apex. The stress on the distal implants was greater than that on the mesial implants; equivalent stress cloud diagrams were also found for all nine maxillary models (Figure 5). There, the stress was mainly concentrated on the cervical margin junction and cortical bone of the distal implants. The stress on the distal bone of the distal implant was greater than that on the mesial bone of the distal implant. Thus, the stress on the distal implant and its distal cortical and cancellous bones are described in more detail below. In addition, although the maximally stressed region of each model was basically the same, the stress intensities (i.e., the heat map values) were different, especially in the third stage.

### 3.2. Stresses on the Distal Implants and Bones in Stage 3 by Model

**Figure 6:**
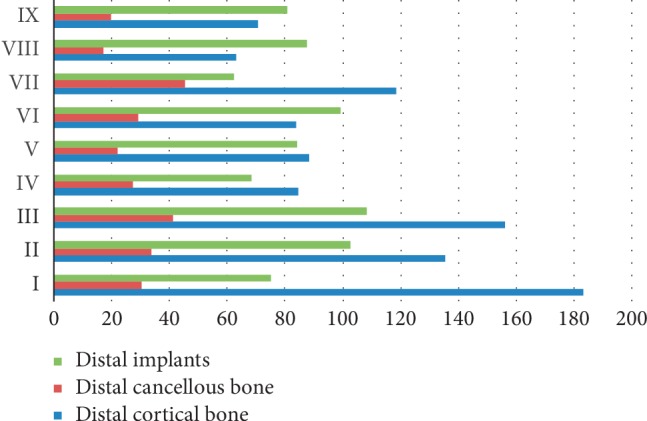
Peak stress in distal implant and surrounding distal bone (MPa). Maximum von Mises stress values are graphed for the distal implants and surrounding bones in dynamic loading stage 3 for the nine models (I–IX, *y*-axis).

The stress levels, according to the masticatory stage, peaked at stage 3, so further analyses will be limited to that stage. In general, the stresses on the distal implants and bones graphed in Figure 6 showed a trend of canine region < lateral incisor region < central incisor region. Only the stresses on the distal cortical and cancellous bones of Models VI, VIII, and IX were lower than that on the distal implant. The stresses on the distal bones were the lowest in Model VIII (62.989 MPa and 17.186 MPa, respectively). The stress on the distal cortical bone was greater than that on the distal implants and cancellous bones in all other models. The maximal stress level was on the distal cortical bone in Model I (183.437 MPa).

### 3.3. von Mises Stress Values in the Nine Maxillary Models

**Figure 7:**
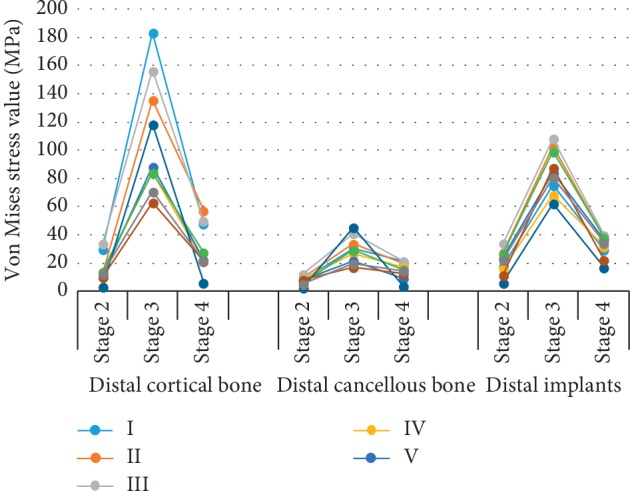
Comparison of maximum von Mises stress values of different parts in different stages of dynamic loading in 9 groups of three-dimensional finite element model of maxilla (MPa).

The von Mises stresses for the nine maxillary 3D finite element models are shown in Table 2 and Figure 7. In each model, the stress values of the distal implants and their distal cortical and cancellous bones over the stages formed an inverted U such that stage 2 < stage 4 < stage 3 stress levels. When ranking the models by mesial implant position, peak stage 3 stress loading in the distal cortical bone was ranked VIII < IX < VI < IV < V < VII < II < III < I, in the distal cancellous bone was VIII < IX < V < IV < VI < I < II < III < VII, and in the distal implants was VII < IV < I < IX < V < VIII <VI < II < III. The stress on the distal implants and the surrounding bone in the central incisor model group was significantly higher than that in the lateral incisor and the canine region model groups; the stress in the canine region was the lowest.

In examining the models, according to the different distal implant tilt angles, when the mesial implants were located in the central incisor region, the stress on the distal cortical bone in stage 3 was ranked 30° (II) <45° (III) <15° (I), and the stresses on the distal cancellous bone and implants were ranked 15° (I) <30° (II) <45° (III). For later incisor mesial implants, the stress on the distal cortical bone was 45° (VI) <15° (IV) <30° (V), the stress on the distal cancellous bone was 45° (VI) <30° (V) <15° (IV) <45° (VI), and that on the distal implants was 15° (IV) <30° (V) <45° (VI). For canine mesial implants, the stresses on the distal cortical bone and distal cancellous bone were ranked 30° (VIII) <45° (IX) <15° (VII), and that on the distal implants was ranked 15° (VII) <45° (IX) <30° (VIII). In addition, the stresses on the distal implants and their distal cortical bones were significantly higher than those on the distal cancellous bones such that the cortical bone values were 2-3-fold higher than those of the cancellous bone.

## 4. Discussion

Misch [22] classified the bone types of the maxillary and mandibular edentulous regions into types D1 (mainly dense cortical bone), D2 (dense, porous ridge crest cortical bone and coarse-grained bone trabecula beneath it), D3 (thin, porous ridge crest cortical bone and fine-grained bone trabecula beneath it), and D4 bone (almost no cortical bone). About 65% of the maxillary anterior edentulous region and 50% of the maxillary posterior edentulous region were composed of the D3-type bone. Misch [22] established a link between brightness on CT, measured in Heinz units, and bone density. Because the CT value of D3-type bone is 350–850 Heinz units, we utilized CT images in this range.

The current 3D finite element method includes static (unchanging) and dynamic (changing over time) loading methods. The dynamic load refers to the load that changes with time in relation to the amount and direction of the external force; this causes the force to generate elastic vibrations or vary in velocity. Masticatory movement is a complex oral reflex activity. Teeth and their supporting tissue are affected by bite force differently in different stages of mastication such that deformation and distribution of stresses on supporting tissue change with the time of mastication. Menicucci et al. [31] discovered that the stress on the implant and its surrounding bone tissue was affected more by the duration, than by the strength, of load. In this study, the masticatory period was divided into five stages; the loading force was equal in each stage, but the loading time, direction, and position changed dynamically. The dynamic loading mode is similar to oral biomechanics and more in line with masticatory movement [19].

In FEA studies to evaluate mechanical stress in the peri-implant bone, it is customary to analyze stresses of various kinds, such as von Mises stress, the maximum/minimum principal stress, and the maximum shear stress. Of these, maximum principal stress is suitable for measuring tensile stress and minimum for compressive stress. However, von Mises stress is the most commonly used scalar-valued stress measure for evaluating the yielding/failure behavior of various materials [32].

In this study, the stress cloud diagrams for the implant models showed that the stress of each model was mainly concentrated in the distal implant necks. The stress of the distal implants was much higher than that of the mesial implants, which suggests that the distal implant neck was a stress concentration zone. However, the intensity and area of the stress concentration regions were different across the models, which indicate that the mesial implant position and the oblique angle of the distal implant affected the stress of the distal implants and the surrounding bone tissue.

The stress cloud diagrams for the bones showed that the stress was mainly concentrated on the junction of the cervical margin and cortical bone of the distal implants. The maximum stress was located in the cortical bone area around the distal implants, and the stress level of cancellous bone was much lower than that of the cortical bone. This supports the previous findings of Koka et al. [33] and Koca et al. [29]. Our results were consistent with the phenomenon that the bone resorption around implants mostly occurs in the cervical cortical bone, and the cancellous bone is less involved. This may be because the stress was concentrated on the implant neck, which is surrounded by the cortical bone. The large stress on the cortical bone thus resulted in bone resorption around the implants.

The results of this study showed that in the 2–4 stages of dynamic loading, the stress of the distal implants and the surrounding bone tissue first increases and then decreases and reached peak stress in the third stage. This indicated that the stress values of each observation point were largest when the loading position was on the lingual slopes of buccal tips of the maxilla. Stage 2 is the initial period of mastication, with short loading time and loading force perpendicular to the occlusal plane; the small contact area results in a small masticatory force. The oblique direction of the loading force and full contact between the upper and lower cusps provided a large contact area and gave full play to the masticatory force in stage 3. Although the loading force was still oblique and the contact area was relatively large in stage 4, about half of the length of the cusp was separated, reducing the stress in the stage. Because the stress in stage 3 was so much greater than the other stages, minimizing stage 3 stress by modeling should minimize bone resorption around the implant.

For models where the implants were located in different regions, the stress of the distal implant and the surrounding bone was the greatest when the implants were located in the central incisor region. It is possible that the stress distribution was inhomogeneous due to the proximity of the two mesial implants and distance from the distal implants. The reduced stress with mesial canine implants was likely due to the more homogeneous stress distribution by the implants on the bone, so setting the implants in the canine area for patients with the maxillary edentulous jaw is the optimal choice.

When modeling the different implant tilt angles in the canine region, the stresses on the distal cortical bone in Models VIII and IX were lesser than those in all other models, and Model VIII was the least, so when implants are located in the canine region, a 30° angle is optimal. Thus, the optimal choice for long-lasting maxillary edentulous jaw implants would be Model VIII, in which the mesial implants are in the canine region and the distal implants are tilted 30°.

Some scholars have pointed out that stress concentration on the marginal bone is the direct cause of bone absorption [34, 35]. This study's results showed that only the stress on the distal cortical and distal cancellous bones in Models VI, VIII, and IX was less than that on the distal implant. Therefore, these models were less likely to cause bone resorption around the implant, especially in Model VIII.

In this study, the models were considered to be 100% osseointegrated, although that is essentially impossible in the actual clinical process. Therefore, some differences between this study's results and clinical practice are likely. However, the dynamic loading method was used to simulate oral masticatory movement more accurately than other static loading models, and this should be carried over into the clinical realm. Further research is recommended to collect more jaw models conforming to the inclusion criteria, which would allow for more 3D finite element models for analysis; longitudinal clinical trials would also provide important data. Our data, however, provide a more reliable clinical basis for dental implant surgery in patients with the edentulous jaw.

## 5. Conclusions

Within the limited scope of this study, the following conclusions can be drawn:

1. The stress of the distal implants is much higher than that of the mesial implants, and the stress of each model is mainly concentrated on the junction of the cervical margin and cortical bone of the distal implants, and the stress level of cancellous bone is much lower than that of the cortical bone. The difference was statistically significant (*P*=0.01, *P* < 0.05).
2. In the temporal stages of dynamic loading, the stress of the distal implants and the surrounding bone tissue first increases and then decreases and peaks in the third stage.
3. The stress of the distal implants and the surrounding bone in the central incisor was significantly higher than that in the lateral incisor and the canine region, and the stress in the canine region was the least. The difference was statistically significant (*P*=0.01, *P* < 0.05).
4. When the mesial implants are located in the canine region and the distal implants tilt at 30°, the stress distribution is lower and more uniform.

## Figures and Tables

**Table 1:** Loading time, direction, position, and loading force at different stages of the masticatory cycle (maxillary posterior teeth).

Loading stage	Loading time (s)	Directions	Position	Loading force (N)
1	0.000∼0.130			
2	0.130∼0.150	Perpendicular to the occlusal plane	Buccal tip, tongue tip	150
3	0.150∼0.260	From the lingual side to the buccal side with 45°	Lingual slopes of the buccal tips	150
4	0.260∼0.300	From the buccal side to the lingual side with 45°	Buccal slopes of the lingual tips	150
5	0.300∼0.875			

**Table 2:** Comparison of maximum von Mises stress values (MPa) in different stages of dynamic loading in nine three-dimensional finite element models of the maxilla.

Model	Distal cortical bone			Distal cancellous bone			Distal implants		
	Stage 2	Stage 3	Stage 4	Stage 2	Stage 3	Stage 4	Stage 2	Stage 3	Stage 4
I	29.891	183.437	48.033	8.277	30.266	21.287	18.127	75.220	29.558
II	12.244	135.625	57.249	10.261	33.747	19.621	27.490	102.563	38.003
III	33.893	156.283	50.033	12.199	41.190	21.106	34.024	108.382	39.914
IV	13.632	84.379	21.473	8.793	27.107	17.300	16.424	68.219	31.880
V	10.522	88.315	22.398	7.793	21.792	9.289	23.797	84.168	36.725
VI	13.942	83.928	27.668	6.211	29.149	15.591	26.534	99.094	38.021
VII	3.270	118.346	6.095	2.653	45.393	3.802	6.028	62.335	16.935
VIII	10.076	62.989	21.373	8.271	17.186	12.709	11.504	87.629	22.278
IX	12.349	70.692	21.751	5.170	19.793	14.689	22.278	80.862	34.266

## Data Availability

The data used to support the findings of this study are included within the article.

## References

[1] Agliardi E. L., Francetti L., Romeo D., Del Fabbro M. (2009). Immediate rehabilitation of the edentulous maxilla: preliminary results of a single cohort prospective study. *International Journal of oral & Maxillofacial Implants*.

[2] Pomares C. (2009). A retrospective clinical study of edentulous patients rehabilitated according to the “all on four” or the “all on six” inmediate function concept. *European Journal of Oral Implantology*.

[3] Agliardi E., Clericò M., Ciancio P., Massironi D. (2010). Immediate loading of full-arch fixed prostheses supported by axial and tilted implants for the treatment of edentulous atrophic mandibles. *Quintessence International*.

[4] Agliardi E., Panigatti S., Clericò M., Villa C., Malò P. (2010). Immediate rehabilitation of the edentulous jaws with full fixed prostheses supported by four implants: interim results of a single cohort prospective study. *Clinical Oral Implants Research*.

[5] Yang J., Xiang H.-J. (2007). A three-dimensional finite element study on the biomechanical behavior of an FGBM dental implant in surrounding bone. *Journal of Biomechanics*.

[6] Maló P., Rangert B., Nobre M. (2003). “All-on-four” immediate-function concept with Branemark system implants for completely edentulous mandibles: a retrospective clinical study. *Clinical Implant Dentistry and Related Research*.

[7] Krekmanov L., Kahn M., Rangert B., Lindström H. (2000). Tilting of posterior mandibular and maxillary implants for improved prosthesis support. *International Journal of Oral & Maxillofacial Implants*.

[8] Soto-Penaloza D., Zaragozí-Alonso R., Penarrocha-Diago M. A., Penarrocha-Diago M. (2017). The all-on-four treatment concept: systematic review. *Journal of Clinical and Experimental Dentistry*.

[9] Sevimay M., Turhan F., Kiliçarslan M. A., Eskitascioglu G. (2005). Three-dimensional finite element analysis of the effect of different bone quality on stress distribution in an implant-supported crown. *The Journal of Prosthetic Dentistry*.

[10] Vafaei F., Khoshhal M., Bayat-Movahed S. (2011). Comparative stress distribution of implant-retained mandibular ball-supported and bar-supported overlay dentures: a finite element analysis. *Journal of Oral Implantology*.

[11] Romeo E., Lops D., Margutti E., Ghisolfi M., Chiapasco M., Vogel G. (2003). Implant-supported fixed cantilever prostheses in partially edentulous arches. A seven-year prospective study. *Clinical Oral Implants Research*.

[12] Meijer H. A., Starmans F. J. M., Steen W. H. A., Bosman F. (1996). Loading conditions of endosseous implants in an edentulous human mandible: a three-dimensional, finite-element study. *Journal of Oral Rehabilitation*.

[13] Yoon K.-H., Kim S.-G., Lee J.-H., Suh S.-W. (2011). 3D finite element analysis of changes in stress levels and distributions for an osseointegrated implant after vertical bone loss. *Implant Dentistry*.

[14] Geng J.-P., Tan K. B. C., Liu G.-R. (2001). Application of finite element analysis in implant dentistry: a review of the literature. *The Journal of Prosthetic Dentistry*.

[15] Ozan O., Kurtulmus-Yilmaz S. (2018). Biomechanical comparison of different implant inclinations and cantilever lengths in all-on-4 treatment concept by three-dimensional finite element analysis. *The International Journal of Oral & Maxillofacial Implants*.

[16] Saber F. S., Ghasemi S., Koodaryan R., Babaloo A., Abolfazli N. (2015). The comparison of stress distribution with different implant numbers and inclination angles in all-on-four and conventional methods in maxilla: a finite element analysis. *Journal of Dental Research, Dental Clinics, Dental Prospects*.

[17] Wang R.-F., Kang B., Lang L. A., Razzoog M. E. (2009). The dynamic natures of implant loading. *The Journal of Prosthetic Dentistry*.

[18] Yoda N., Liao Z., Chen J., Sasaki K., Swain M., Li Q. (2016). Role of implant configurations supporting three-unit fixed partial denture on mandibular bone response: biological-data-based finite element study. *Journal of Oral Rehabilitation*.

[19] Hajimiragha H., Abolbashari M., Nokar S., Abolbashari A., Abolbashari M. (2014). Bone response from a dynamic stimulus on a one-piece and multi-piece implant abutment and crown by finite element analysis. *Journal of Oral Implantology*.

[20] Ozkir S. E., Unal S. M., Yurekli E., Güven S. (2016). Effects of crown retrieval on implants and the surrounding bone: a finite element analysis. *The Journal of Advanced Prosthodontics*.

[21] Zhang G., Yuan H., Chen X. (2016). A three-dimensional finite element study on the biomechanical simulation of various structured dental implants and their surrounding bone tissues. *International Journal of Dentistry*.

[22] Misch C. E., Li D. H. (2015). *Modern Dental Implant*.

[23] Bulaqi H. A., Mousavi Mashhadi M., Safari H., Samandari M. M., Geramipanah F. (2015). Effect of increased crown height on stress distribution in short dental implant components and their surrounding bone: a finite element analysis. *The Journal of Prosthetic Dentistry*.

[24] Topkaya T., Solmaz M. Y., Dundar S., Eltas A. (2014). Numerical analysis of the effect of implant geometry to stress distributions of dental implant system. *Cumhuriyet Dental Journal*.

[25] Munari L. S., Cornacchia T. P. M., Moreira A. N., Gonçalves J. B., De Las Casas E. B., Magalhães C. S. (2015). Stress distribution in a premolar 3D model with anisotropic and isotropic enamel. *Medical & Biological Engineering & Computing*.

[26] Xin P., Nie P., Jiang B. (2013). Material assignment in finite element modeling: heterogeneous properties of the mandibular bone. *Journal of Craniofacial Surgery*.

[27] Horita S., Sugiura T., Yamamoto K., Murakami K., Kirita T. (2017). Biomechanical analysis of immediately loaded implants according to the “all-on-four” concept. *International Journal of Oral and Maxillofacial Surgery*.

[28] Benzing U. R., Gall H., Weber H. (1995). Biomechanical aspects of two different implant-prosthetic concepts for edentulous maxillae. *The International Journal of Oral & Maxillofacial Implants*.

[29] Koca O. L., Eskitascioglu G., Usumez A. (2004). Three-dimensional finite-element analysis of functional stresses in different bone locations produced by implant placed in the maxillary posterior region of the sinus floor. *The Journal of Prosthetic Dentistry*.

[30] Li H. B., Yao Y. L. (2007). The dynamic characters of bite force changing in laterotrusions. *Chinese Journal of Prosthodontics*.

[31] Menicucci G., Mossolov A., Mozzati M., Lorenzetti M., Preti G. (2002). Tooth-implant connection: some biomechanical aspects based on finite element analyses. *Clinical Oral Implants Research*.

[32] Shigemitsu R., Yoda N., Ogawa T. (2014). Biological-data-based finite-element stress analysis of mandibular bone with implant-supported overdenture. *Computers in Biology and Medicine*.

[33] Koka P., Mohapatra A., Anandapandian P. A., Murugesan K., Vasanthakumar M. (2012). The effect of implant design on the stress distribution in a three-unit implant-supported distal cantilever fixed partial denture: a three-dimensional finite-element analysis. *Indian Journal of Dental Research*.

[34] Baggi L., Cappelloni I., Di Girolamo M., Maceri F., Vairo G. (2008). The influence of implant diameter and length on stress distribution of osseointegrated implants related to crestal bone geometry: a three-dimensional finite element analysis. *The Journal of Prosthetic Dentistry*.

[35] Misch C. E., Suzuki J. B., Misch-Dietsh F. M., Bidez M. W. (2005). A positive correlation between occlusal trauma and peri-implant bone loss: literature support. *Implant Dentistry*.

